# Comment on Jones et al. Application of a Novel Algorithm for Expanding Newborn Screening for Inherited Metabolic Disorders across Europe. *Int. J. Neonatal Screen.* 2022, *8*, 20

**DOI:** 10.3390/ijns9010007

**Published:** 2023-02-15

**Authors:** Rose Maase, Marleen E. Jansen, Marie-Louise Heijnen, Eugenie Dekkers

**Affiliations:** 1 Centre for Health Protection, National Institute for Public Health and the Environment (RIVM), 3720 BA Bilthoven, The Netherlands; 2 Centre for Population Screening, National Institute for Public Health and the Environment (RIVM), 3720 BA Bilthoven, The Netherlands

With innovations in both the screening methodologies and treatment of diseases, newborn screening (NBS) programmes are confronted with an increasing number of candidate diseases. Given the time and resources required for well-substantiated and coordinated expansion, deciding which diseases to add to a programme is challenging. This is certainly the experience of the Dutch NBS programme, which is why we read the article “Application of a Novel Algorithm for Expanding Newborn Screening for Inherited Metabolic Disorders across Europe” with great interest [1]. The authors provide a logical and well-justified matrix with which to prioritize candidate diseases, the aspects of which we plan to apply in the establishment of our own resources for prioritization and not only for inborn errors of the metabolism, but for all of our candidate diseases.

We are developing a broader set of scoring criteria, which will be suitable for application to a comprehensive range of diseases, and which will reflect our current programme. For example, based on the experience from recent programme expansions, we intend to add a criterion for ‘target disease definition’. This criterion differentiates between a disease for which the target disease can be clearly defined *and* detected during NBS before referral for clinical confirmation, and a disease with a broad spectrum of phenotypes, such as infantile, juvenile and adult-onset ones, which cannot be differentiated within the screening setting. The former would score higher on this criterion.

Furthermore, in the ‘Testing Strategy’, the availability of a CE-IVD kit will weigh heavily in favour for a disease when it is compared to diseases for which laboratory developed tests (LDT) are required: additional points will be awarded for each tier for which a CE-IVD kit is available. This is in part due to the recent implementation of the Medical Devices Act, which requires that commercially available test kits should be used if they are available and suitable for the purpose. Additionally, the complexities associated with using an LDT for nationwide screening, such as obtaining nationwide uniformity and the consistent quality of measurements across our five screening laboratories or the logistics of the transportation of samples when a single laboratory conducts an LDT for all the samples, make LDTs a less desirable option. Extra points might be awarded if the expanded use of a CE-IVD multiplex test kit already in use within our programme will facilitate the screening.

We also plan to incorporate a set of ‘knock-out’ criteria; diseases which fulfil such criteria will still receive a points-based ranking, but they will be placed on a separate list. For example, diseases for which there is no suitable screening test method for detection (CE-IVD or LDT) or for which there is no available and approved treatment will be listed separately. Diseases which fulfil one or more of the ‘knock-out’ criteria will then be re-evaluated only once the unfulfilled criterion has been satisfied or a suitable alternative becomes available.

By broadening the scoring criteria, adapting it to our own programme and establishing the ‘knock-out’ criteria, we aim to establish a point-based ranking for each candidate disease, which is tailor made for our current programme and which also reflects our aspiration to develop a sustainable and flexible NBS programme in the future. Moreover, by discussing this with our stakeholders, we hope to achieve even more transparency about our plans.

We thank the authors for their inspiring and thought-provoking article and would like to take this opportunity to bring several inaccuracies to their attention regarding the Dutch NBS programme. As the authors mention in the discussion, severe combined immunodeficiency was added to our NBS programme in January 2021 following a regional pilot, which began in April 2018. This is, however, not reflected in Appendix A. Furthermore, without regional pilots propionic acidemia, methylmalonic acidemia and carnitine palmitoyltransferase deficiency type 1 were added to our programme in October 2019, and mucopolysaccharidosis type I was added in March 2021, respectively, four and a half and six years after the recommendation for their inclusion. Homocystinuria was included in our programme from 2007 to 2010 (it was removed because of a high false positive rate and there being no true positives in this period). Adrenoleukodystrophy (ALD) is not included in our programme, but a regional pilot was conducted in 2021, and ALD will be considered for addition to the Dutch NBS programme once a review of the pilot has been completed.

We would be grateful if these inaccuracies could be rectified such that the status of the Dutch screening programme at the time of publication is accurately described.

## References

[1] Jones S.A., Cheillan D., Chakrapani A., Church H.J., Heales S., Wu T.H.Y., Morton G., Roberts P., Sluys E.F., Burlina A. (2022). Application of a Novel Algorithm for Expanding Newborn Screening for Inherited Metabolic Disorders across Europe. Int. J. Neonatal Screen..

